# Structural Geometry Variation of 1,4-Naphthalene-Based Co-Polymers to Tune the Device Performance of PVK-Host-Based OLEDs

**DOI:** 10.3390/polym13172914

**Published:** 2021-08-30

**Authors:** Qian Liu, Dhanashree Moghe, Gopa Sardar, Sergei Manzhos, Steven E. Bottle, Aung Ko Ko Kyaw, Dinesh Kabra, Prashant Sonar

**Affiliations:** 1 Centre for Materials Science, Queensland University of Technology, Brisbane, QLD 4000, Australia; leuking0921@163.com (Q.L.); s.bottle@qut.edu.au (S.E.B.); 2 Guangdong University Key Laboratory for Advanced Quantum Dot Displays, Shenzhen Key Laboratory for Advanced Quantum Dot Displays and Lighting, and Department of Electrical and Electronic Engineering, Southern University of Science and Technology, Shenzhen 518055, China; aung@sustech.edu.cn; 3 Department of Physics, Indian Institute of Technology Bombay, Mumbai 400076, India; damoghe@gmail.com (D.M.); gopa.phy@iitb.ac.in (G.S.); 4 Centre Énergie Matériaux Télécommunications, Institut National de la Recherche Scientifique, 1650 boulevard Lionel-Boulet, Varennes, QC J3X1S2, Canada; sergei.manzhos@gmail.com

**Keywords:** structural geometry, 1,4-naphthalene, phenothiazine, triphenylamine substituted fluorene, anthanthrene, polymers, poly(9-vinyl carbazole), organic light-emitting diodes

## Abstract

Blue-color-emitting organic semiconductors are of significance for organic light-emitting diodes (OLEDs). In this study, through Suzuki coupling polymerization, three 1,4-naphthalene-based copolymers—namely, PNP(1,4)-PT, PNP(1,4)-TF, and PNP(1,4)-ANT—were designed and synthesized. The variation of comonomers, phenothiazine (PT), triphenylamine substituted fluorene (TF), and anthanthrene (ANT), effectively tuned the emitting color and device performance of poly(9-vinyl carbazole) (PVK)-based OLEDs. Especially, the polymer PNP(1,4)-TF, bearing perpendicular aryl side groups, showed a most twisted structural geometry, which enabled an ultra-high thermal stability and a best performance with blue emitting in PVK-host-based OLEDs. Overall, in this work, we demonstrate a promising blue-color-emitting polymer through structural geometry manipulation.

## 1. Introduction

Organic semiconducting materials have multiple applications in organic electronics and optoelectronics [1,2,3,4,5]. Organic light-emitting diodes (OLEDs) are one of the most promising candidates for flexible displays and lighting applications [6,7,8,9,10,11,12]. In an OLED device, the light-emitting material is a crucial component where the holes and electrons recombine to emit light [8]. For full-color displays, three primary colors should be included, i.e., red, blue, and green. Thereinto, the development of blue-light-emitting polymers greatly lags behind the two others [13,14]. Naphthalene, with an acene structure, is one of the most widely used building blocks to construct conjugated materials for blue-color OLEDs [15,16]. However, the repeating units of naphthalene-based copolymers are mainly naphthalene derivatives, including binaphthalene, naphthylenevinylene, and alkoxy naphthalene, etc. For example, Samanta et al. reported that 1,4-polynaphthalene is a promising polymer to fabricate true color blue OLEDs through solution-processed technique [17]. The building blocks of 1,4-polynaphthalene are naphthalene and alkoxy naphthalene, respectively. It is rarely reported that copolymers with naphthalene and other type moieties (non-naphthalene derivatives) are used as blue-emitting materials in OLEDs.

For the selection of comonomers, those with non-planar structural geometries are promising, which tends to endow the resultant polymers with twisted backbones to decrease the molecular aggregation, and consequentially, to avoid fluorescence quenching [18]. Phenothiazine (PT) has a butterfly-like structure that enables its non-planar geometry [19,20]. Additionally, aryl groups attached on the N atoms are highly twisted relative to the PT unit [21]. For polymers, the 3,7-position active sites contribute to the formation of a flexural mainchain, also leading to distortion between PT and the comonomer [22,23]. All these features demonstrate the great potential of PT-based materials in OLEDs. Fluorene is a popular building block in the design of materials for OLED devices; however, its backbone planarity is a disadvantage [24]. Klaus Müllen et al. introduced triphenylamine (TPA) side groups to fluorene, delivering a new building block, i.e., TPA-substituted fluorene (TF) [25,26]. The ultra-twisted TPA units (with an around 90-degree dihedral angle to the backbone) allow its homopolymer (poly-TF) a very good solubility, providing the possibility of device fabrication via solution processable techniques [26]. The OLED device fabricated with poly-TF through the spin-coating method showed a pure blue emission, demonstrating the huge potential of TF to construct emissive materials for OLEDs. Another building block we are interested in is the anthanthrone (ANT), which is a very promising building block in organic electronics because of its ultra-large fused-ring π structure [27,28]. In our group, we have developed ANT-based materials for various electronic devices, including perovskite solar cells, organic photovoltaic devices, thin film transistors, and chemical sensors [27,29]. It is reported that the substitutions on different active sites of ANT also enable highly efficient blue-color OLEDs [30], prompting us to study ANT-based polymers for OLEDs.

Herein, we developed three new polymers by copolymerizing 1,4-naphthalne with the three building blocks mentioned above. They are named as PNP(1,4)-PT, PNP(1,4)-TF, and PNP(1,4)-ANT. The three polymers have different structural geometries, the effects of which on their thermal, optical, and electrochemical properties are studied. Moreover, pristine polymer-based and host–guest blend-based OLED devices were fabricated to study their electroluminescence performance.

## 2. Experimental Section

**Scheme 1:**
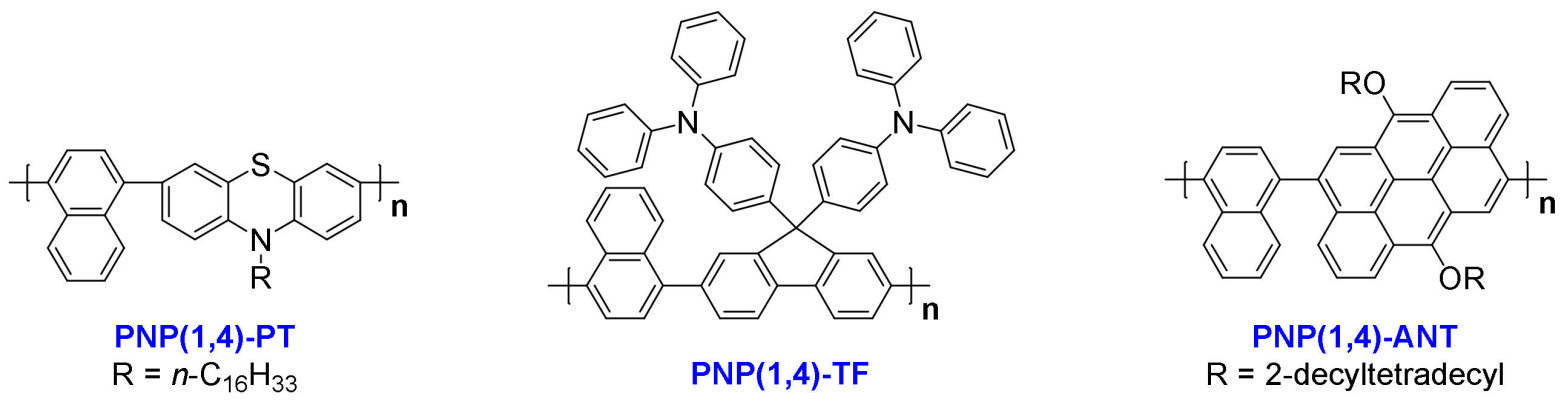
Chemical structures of the three polymers studied in this work. The detailed synthetic routes and procedures are shown in the Supplementary Materials.

Unless otherwise stated, the starting compounds are commercially available and were used directly without further purification. The chemical structures of the three polymers studied in this work are shown in Scheme 1. The detailed synthetic routes, procedures and characterizations of monomers and polymers are shown in the Supplementary Materials.

General Procedure of Polymerization: To a 50 mL Schlenk flask, two monomers (0.25 mmol each) are dissolved in anhydrous toluene (10 mL). A portion of 2 M K_2_CO_3_ aqueous solution (5 mL) and 2 drops of Aliquat 336 are added in the above reaction mixture. The solution is purged with argon for 30 min, followed by the addition of Pd(PPh_3_)_4_ (11 mg, 0.01 mmol). The reaction is stirred at 80 °C for around 3 days. Then, 4-methoxyphenylboronic acid and 2-bromothiophene are separately added with a time interval of around 30 min. After stirring overnight, the resulting mixture is poured into a mixture of methanol (100 mL) and water (100 mL). The precipitated solid is filtered off and subjected to Soxhlet extraction with methanol (24 h), acetone (24 h), and hexane (24 h), respectively. The residue is extracted with chloroform, further purified by running a fast column, and finally precipitated again from methanol, obtaining the target polymers. PNP(1,4)-PT (112 mg, 81.5%): Molecular weight: Mn = 9.059 kDa, Mw = 14.76 kDa, PDI = 1.63; PNP(1,4)-TF (154 mg, 79.1%): Molecular weight: Mn = 13.23 kDa, Mw = 33.94 kDa, PDI = 2.56; PNP(1,4)-ANT (232 mg, 83.8%): Molecular weight: Mn = 72.98 kDa, Mw = 330.2 kDa, PDI = 4.52.

OLED Devices Fabrication: The devices are prepared with the structure of ITO/PEDOT:PSS/emitter or emitter blend/Ca/Al. Glass substrates with patterned indium tin oxide (ITO) are cleaned with soap, deionized water, acetone, and isopropanol in ultrasonic bath sequentially for 10 min each. The substrates are then treated with oxygen plasma and spin-coated with the hole injection layer of poly(3,4-ethylenedioxythiophene): polystyrene sulfonate (PEDOT:PSS) (from *Clevios*) at 5000 rpm for 60 s, followed by annealing at 150 °C under nitrogen for 30 min. The samples are then transferred to a nitrogen-filled glove box. The emitter (each of the three polymers) and PVK are dissolved separately in chlorobenzene with a concentration of 5 mg mL^−1^ and 10 mg mL^−1^, respectively. The solutions are magnetically stirred to become clear. Pristine films are prepared by spin-coating the emitter solution at 1500 rpm for 50 s. For blend films, the two solutions are mixed in the desired ratio (6 wt% to 75 wt% of emitter: PVK) and then spin-coated onto the annealed PEDOT:PSS layer at 1500 rpm for 50 s. The blend films are annealed gradually from 130–180 °C under inert condition for around 40 min. The samples are transferred to a thermal evaporation chamber integrated with a glovebox for Ca/Al deposition under the vacuum of 1 × 10^−6^ mbar and then hermetically sealed before characterization.

Time-Correlated Single Photon Counting: The time-correlated single photon counting (TCSPC) is done on Horiba Jobin Yvon IBH, UK, with 10^−6^ M solution in *p*-xylene with the time resolution of 7 ps. For TCSPC, PNP(1,4)-PT and PNP(1,4)-TF are excited with a 375 nm laser, having the instrument response function (IRF) of ~180 ps. The photoluminescence (PL) decay was measured at 471 nm and 430 nm for PNP(1,4)-PT and PNP(1,4)-TF, respectively. The fitting of the PL decay was done using the monoexponential decay equation of IBH DAS v6.2 data analysis software.

## 3. Results and Discussion

### 3.1. Design and Synthesis

Naphthalene-based polymers are some of the most suitable materials for OLED applications. Among the several reactive sites, 1,5- or 2,6-naphthalene-contained polymers are widely reported, while 1,4-naphthalene-based materials are rarely studied [17]. Additionally, by selecting different comonomers with non-planar structures, not only the bandgaps of resultant polymers are varied to tune the emitting color of fabricated OLEDs, but also the polymer backbone geometries tend to be twisted to avoid strong molecular aggregation and thus decrease the fluorescence quenching in OLEDs. Herein, we designed and synthesized three new polymeric materials based on 1,4-naphthalene through Suzuki coupling polymerization with N-alkylated phenothiazine (PT), triphenylamine substituted fluorene (TF), and alkoxy anthanthrene (ANT), respectively. The dibromo compounds DB-PT, DB-TF, and DB-ANT (see Supplementary Materials for the structures) were synthesized by following reported procedures [26,31,32]. The boronic ester compounds BE-NP(1,4) and BE-PT (See Supplementary Materials for the structures) were synthesized by using the well-known procedure in the presence of bis(pinacolato)diboron, Pd(dppf)Cl_2_, and KOAc in N,N-Dimethylformamide (DMF) at 90 °C for 24 h. Finally, Suzuki coupling reactions were conducted in the presence of Pd(PPh_3_)_4_, K_2_CO_3_ aq. (2 M), and Aliquat 336 in anhydrous toluene at 80 °C for around 3 days to get the three target polymers. The polymers were purified through Soxhlet extraction, and their molecular weights were measured by gel permeation chromatography (GPC) under nitrogen atmosphere at ambient temperature.

### 3.2. Thermal Properties

The thermal properties of three polymers were measured through thermogravimetric analysis (TGA) and differential scanning calorimetric (DSC) analysis. As shown in Figure S1a–c in the Supplementary Materials, their decomposition temperatures (T_d_, 5% weight loss) were 372 °C for PNP(1,4)-PT, 549 °C for PNP(1,4)-TF and 269 °C for PNP(1,4)-ANT. Overall, the thermal stability of all three polymers was sufficient for possible practical applications and thermal annealing treatment as required in device performance optimization. Notably, PNP(1,4)-TF had a quite high thermal stability with the T_d_ value of higher than 500 °C. That was due to the highly rigid structure in both the main chain and side chain backbones, which is favorable for stable OLEDs [26]. From the DSC curves as shown in Figure S1d under heating and cooling processes between 25 °C and 250 °C, no obvious transition peaks were observed, which is common for materials with rigid mainchain skeletons since they generally start to decompose before any thermal transition [33].

### 3.3. Optical Properties

**Figure 1:**
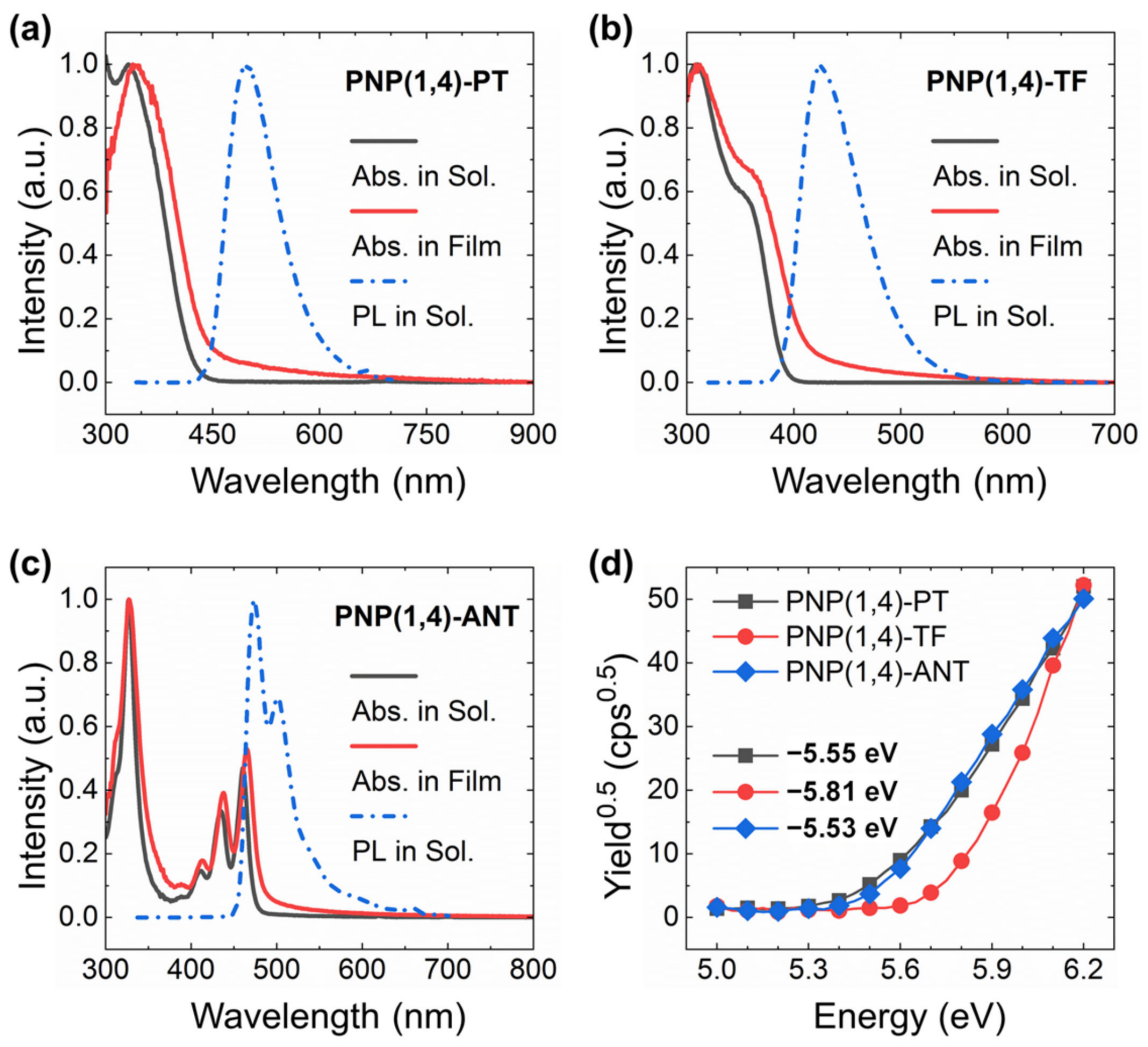
The UV-Vis absorption and PL spectra of (**a**) PNP(1,4)-PT, (**b**) PNP(1,4)-TF, and (**c**) PNP(1,4)-ANT. (**d**) PESA measurements.

The optical properties of the three polymers were studied by ultraviolet visible (UV-Vis) absorption and photoluminescence (PL) spectra as shown in Figure 1a–c. The extracted peak values are summarized in Table 1. All three polymers showed a dominant absorption maximum at around 310–330 nm. This was ascribed to the π–π* transition. PNP(1,4)-TF exhibited an additional shoulder peak at around 363 nm, which is the S0→S1 transition and is very close to the computed value (as confirmed by DFT simulations, see Figure S3 and Table S1 in Supplementary Materials). PNP(1,4)-ANT displayed the typical feature of anthanthrene-based materials because of the large conjugated π structure where the π-electrons are localized on two directions, 4,10- (alkoxy direction) and 6,12- (polymeric direction) positions [30,34]. The polymers’ film spectra exhibited only a slight red shift in comparison with the solution spectra, demonstrating the minor degree of intermolecular aggregation in solid state. This is a favorable feature for OLED application since molecular aggregation often quenches the emission. From the onset wavelengths of film spectra, the bandgaps of three polymers were calculated to be 2.82, 3.00, and 2.57 eV, respectively, as summarized in Table 1. Obviously, different comonomers introduction caused variant bandgaps, which are expected to emit different colors in OLEDs. Excited at the wavelength of absorption peak of each polymer, the obtained PL spectra displayed a maximum emission peak at 496, 426, and 474 nm for PNP(1,4)-PT, PNP(1,4)-TF, and PNP(1,4)-ANT, respectively, which confirms their variant emissive color, as shown in Figure S2 in Supplementary Materials.

### 3.4. Energy Levels Estimation

The highest occupied molecular orbital (HOMO) levels of three polymers were determined through photoelectron spectroscopy in air (PESA) by depositing the polymer films on glass substrates. Extracted from the PESA curves in Figure 1d, the HOMO values were estimated to be −5.55, −5.81, and −5.53 eV for PNP(1,4)-PT, PNP(1,4)-TF, and PNP(1,4)-ANT, respectively. Since the lowest unoccupied molecular orbital (LUMO) levels were not obtainable from PESA measurement, their values were calculated by considering the optical bandgap of each polymer using the equation of E_LUMO_ = E_HOMO_ + E_g_^opt.^. They were calculated to be −2.73, −2.81, and −2.96 eV for PNP(1,4)-PT, PNP(1,4)-TF, and PNP(1,4)-ANT, respectively. In OLEDs, the introduction of host–guest blend in the active layer can effectively enhance the device performance and operational stability [35]. PVK is one of the typical host materials, the energy levels of which are −5.81 eV (HOMO) and −2.20 eV (LUMO). Simply considering the energy levels, the addition of each polymer (as the guest material) into PVK can effectively enhance/retain holes injection efficiency. The LUMO of PNP(1,4)-ANT was lower than the work function of the cathode −2.90 eV for Ca/Al in the current case, which may cause decreasing of electrons injection efficiency, leading to poor device performance.

### 3.5. Density Functional Theory (DFT) Calculations

**Figure 2:**
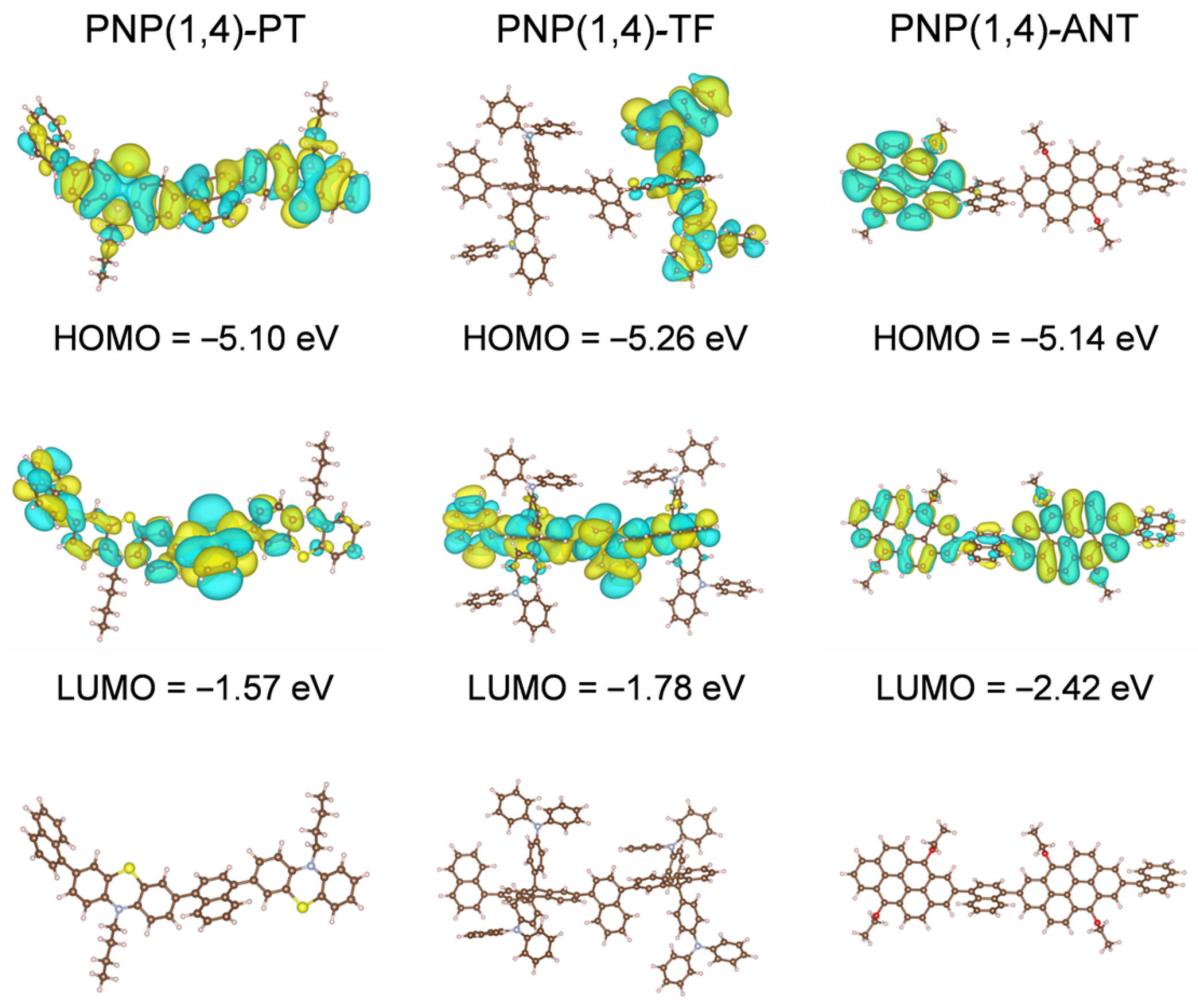
The DFT computed HOMO-LUMO orbital distributions and the front-view geometries of polymer dimers with shortened side chains for simplicity.

DFT calculations were performed to study the electronic structures of the three polymers. Polymer dimers with shortened alkyl side chains were used as the representatives for simplicity. As shown in Figure 2, the HOMO orbitals of PNP(1,4)-TF and PNP(1,4)-ANT were delocalized on the TF and ANT units, respectively, while it spread over the entire backbone in the case of PNP(1,4)-PT, indicating the weaker electron-donating property of PT. It is worth noting that the TPA units of TF also contributed to the HOMO orbital coupling, explaining the lowest HOMO level of PNP(1,4)-TF among all three polymers. The electron density of LUMO was distributed over the whole main chain backbone in all cases, which is beneficial for efficient electrons transport [35]. The computed HOMO and LUMO values of all three polymers followed the same trend as the experimental results. The higher absolute HOMO and LUMO levels in DFT calculations were reasonable as they did not include the effects of broadening and approximations, which were included in the experimental estimations according to the measured PESA results and optical bandgaps [36,37].

The front-view geometries indicate that the dihedral angels of polymer dimers were 56°, 56°, and 59° for PNP(1,4)-PT, PNP(1,4)-TF, and PNP(1,4)-ANT, respectively. This twisted backbone structure is good for OLED applications owing to minimum emission quenching in solid state. Although the dihedral angles in the backbone direction of all three polymers were nearly identical, the TPA units of PNP(1,4)-TF were found to be orthorhombic to the polymer main chain, making it the most-twisted overall structural geometry. In addition, the butterfly-like structure of PT unit and the 3,7-position reactive sites led to a more flexural polymer main chain for PNP(1,4)-PT than PNP(1,4)-ANT. The differences of structural geometries of all three polymers are believed to influence the molecular packing and the miscibility with the PVK host in OLED devices, and thus result in variant device performance. The computed absorption and emission spectra are shown in Figure S3 in Supplementary Materials and the extracted data and calculated Stokes shifts are summarized in Table S1. It is clear that PNP(1,4)-TF displays the smallest Stokes shift from the DFT calculation. From the experimental results, PNP(1,4)-TF was calculated to have a Stokes shift of 64 nm, which is higher than that of PNP(1,4)-ANT. However, the shoulder peak in PL spectrum of PNP(1,4)-ANT indicates its relatively strong molecular packing which is unfavorable for OLED devices.

### 3.6. OLED Device Performance and Discussion

**Figure 3:**
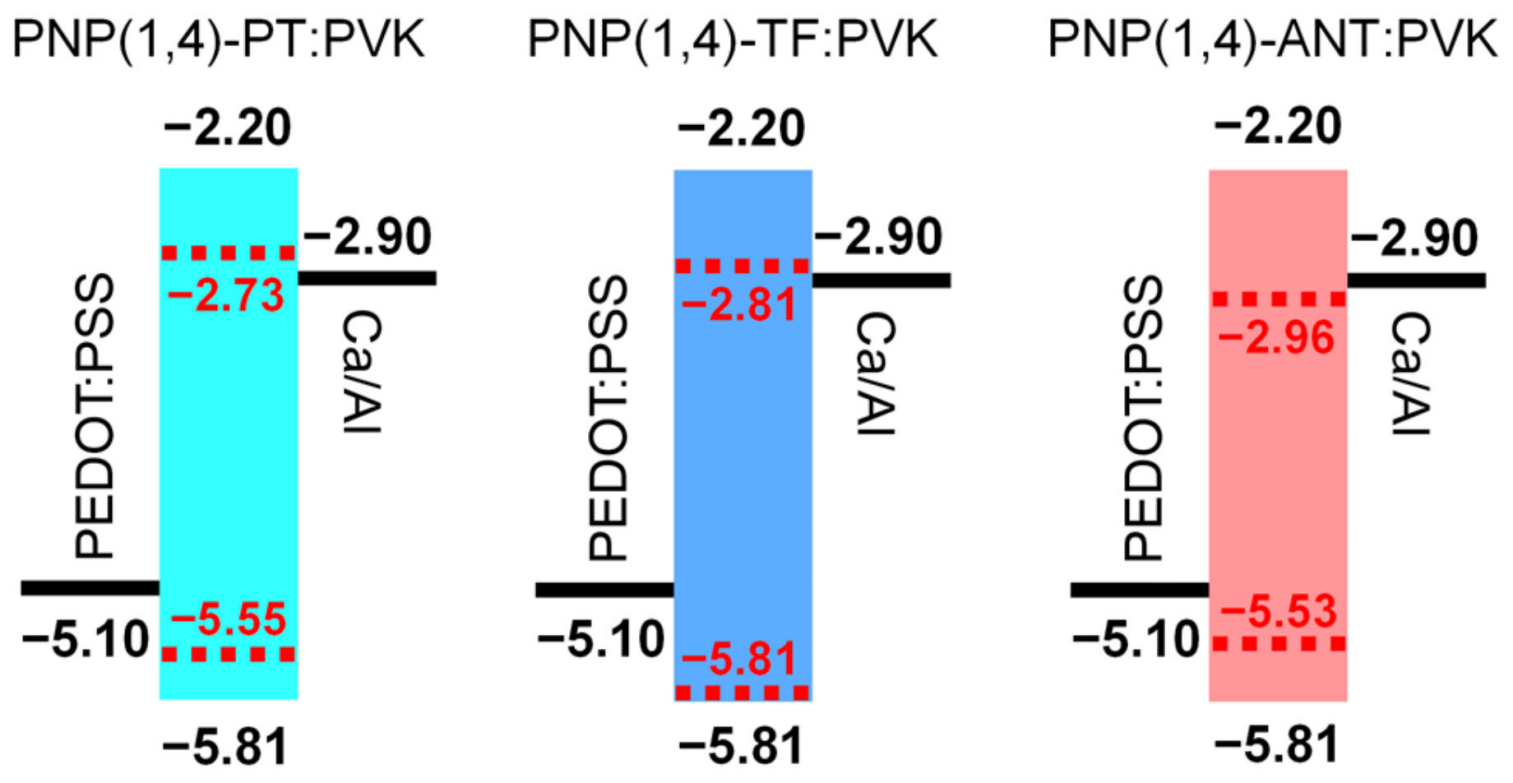
Energy level band diagram of PEDOT:PSS/Polymer:PVK/Ca/Al.

Due to the intense luminescence of all three polymers observed under laboratory UV light (Figure S2), OLED devices were fabricated to further study their electroluminescence (EL) property. In the emitting layer of OLED devices, pristine polymer films or blends with PVK host (with different wt% of polymer) were both applied to measure the device performance. The energy level band diagram of each component in the structure of devices is shown in Figure 3. Note that the performance of PNP(1,4)-ANT film or blend with PVK host was too low to obtain valid data; therefore, in this section, we skip the discussion about this polymer.

#### 3.6.1. PNP(1,4)-PT Polymer Results

**Figure 4:**
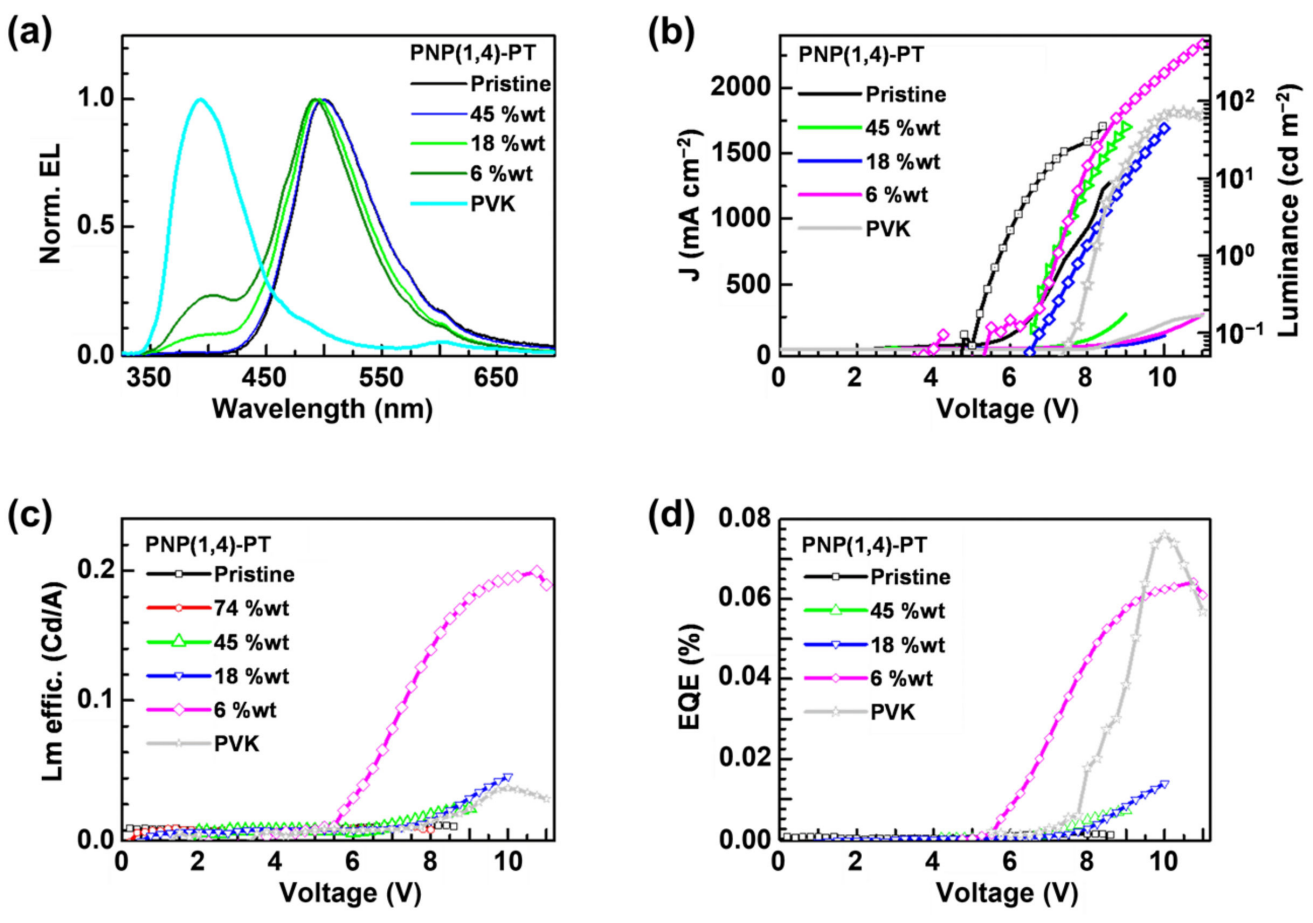
The OLED devices (PEDOT:PSS/PNP(1,4)-PT:PVK/Ca/Al) characterization results: (**a**) EL spectra; (**b**) the J–V (left axis) curves and the luminance (right axis); (**c**) luminance efficiency; and (**d**) external quantum efficiency (EQE).

The normalized EL spectra of PNP(1,4)-PT:PVK blends are shown in Figure 4a, and the images of working OLEDs fabricated with pristine PNP(1,4)-PT or 75 wt% PNP(1,4)-PT:PVK blend are shown in Figure S4. It is observed that the devices fabricated with pristine PNP(1,4)-PT or PNP(1,4)-PT:PVK blend exhibit a blue-green emission. In Figure 4a, the 6 wt% PNP(1,4)-PT:PVK blend shows a EL peak at ~500 nm that is nearly identical to the pristine PNP(1,4)-PT, along with a quenching of the PVK at ~400 nm. As the concentration of PNP(1,4)-PT increases from 0 to 45%, a weak redshift is observed in the EL spectra. With concentrations of higher than 45%, no significant variation in the EL spectra is observed. This demonstrates that the emission of devices based on PNP(1,4)-PT:PVK originated mainly from the PNP(1,4)-PT polymer even at a very low weight ratio. Figure 4b plots the J–V (left axis) curves of the devices, which exhibit a shift to the lower turn-on voltage with the increasing weight concentration of PNP(1,4)-PT. This indicates that the addition of PNP(1,4)-PT guest effectively enhanced the charge carrier transport of devices owing to the more matched energy levels alignment. In the same figure, the luminance is plotted on the right axis. The data from the graph are tabulated in Table S2 in Supplementary Materials. The 6 wt% device reached a maximum luminance of ~600 cd m^−2^ at ~11 V. The ratio of the luminance to the current density (luminance efficiency) and the EQE of the same set of devices are calculated and plotted in Figure 4c,d, respectively. The EQE of the 6 wt% PNP(1,4)-PT:PVK device exhibited a comparable performance to that of the pristine PVK, but the luminance efficiency was enhanced by about 7 times. This result indicates that a small weight percentage of PNP(1,4)-PT in PVK could effectively improve the luminance of the OLED device.

To evaluate the role of film morphology on device performance, atomic force microscope (AFM) studies were performed on the samples with the same weight percentage concentration as was used for devices fabrication (Figure S5). The film roughness values are tabulated in Table S3. It is clear that both the pristine PVK and PNP(1,4)-PT have a low roughness of <1.0 nm. After mixing the PVK and PNP(1,4)-PT, even at a very low weight percentage, the film roughness has a dramatic increment, which is the result of the larger phase separation domains. Observed from the AFM images in Figure S5, the 6 wt% PNP(1,4)-PT:PVK blend has a much better miscibility than others, explaining its much higher emitting performance in the OLED devices.

#### 3.6.2. PNP(1,4)-TF Polymer Results

**Figure 5:**
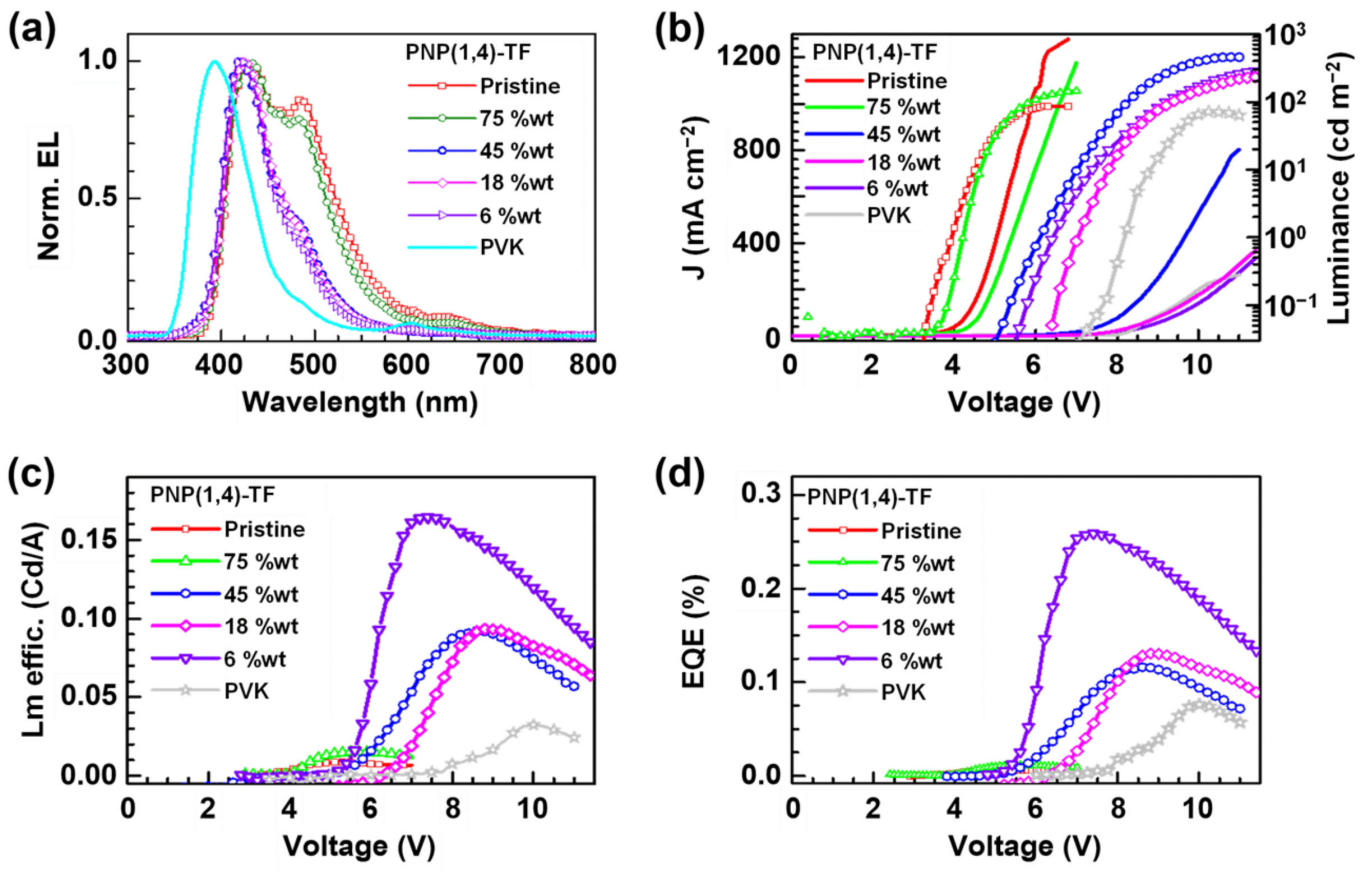
The OLED devices (PEDOT:PSS/PNP(1,4)-TF:PVK/Ca/Al) characterization results: (**a**) EL spectra; (**b**) the J–V (left axis) curves and the luminance (right axis); (**c**) luminance efficiency; and (**d**) external quantum efficiency (EQE).

The normalized EL spectra of pristine PVK, pristine PNP(1,4)-TF, and polymer-PVK blends (with different weight ratios of PNP(1,4)-TF) are shown in Figure 5a. The real working OLED device images based on pristine PNP(1,4)-TF, PVK, or 75 wt% PNP(1,4)-TF:PVK blend are shown in Figure S6 for a clear comparison. It is observed that the addition of PNP(1,4)-TF to PVK effectively enhanced the brightness of resultant devices in comparison with pristine PNP(1,4)-TF and changed the emitting color relative to pristine PVK. Visibly, blue-color OLEDs with a high brightness can be achieved from the PNP(1,4)-TF:PVK blend.

**Figure 6:**
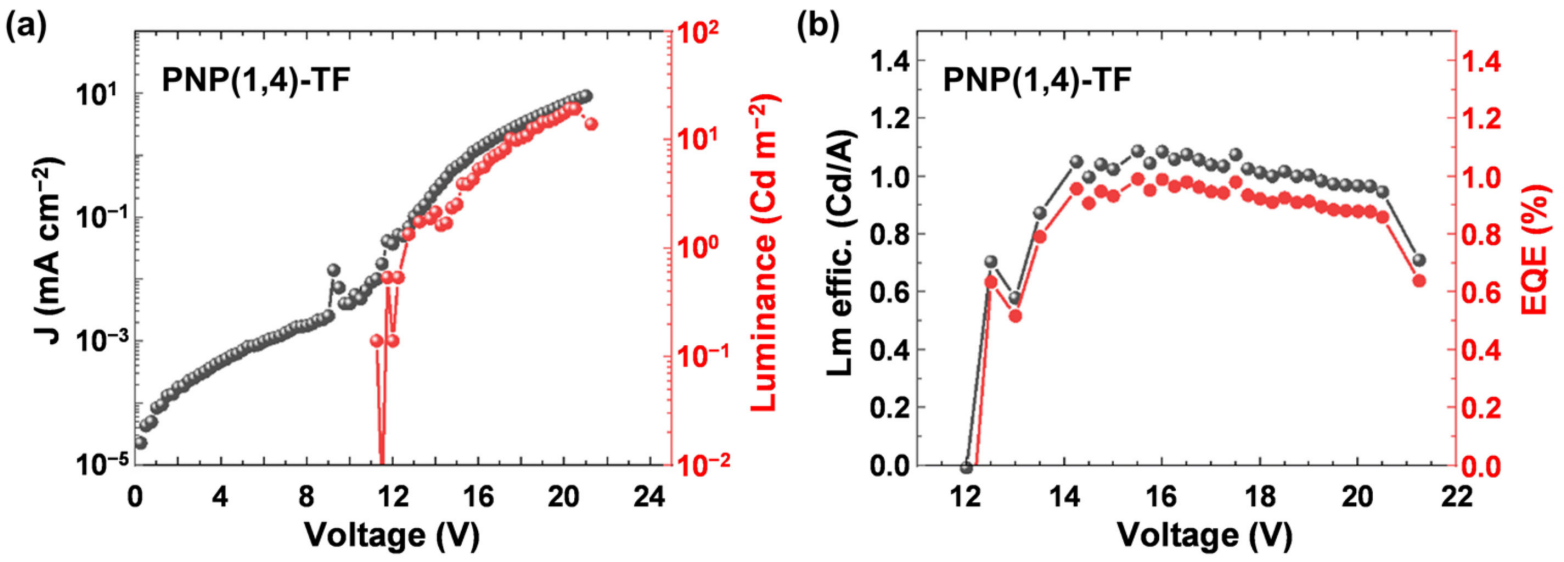
(**a**) Current density–Voltage–Luminance characteristics and (**b**) Luminance efficiency and external quantum efficiency (EQE) of the ITO/PEDOT:PSS/PNP(1,4)-TF:PVK/TPBi/LiF/Al OLED with 6 wt% polymer doped in PVK.

The emission peak of PVK was maximum at ~400 nm, while the addition of PNP(1,4)-TF redshifted the EL spectra. At a lower wt% of the emitting polymer (6–45%), the peaks were located at ~450 nm, while at a higher concentration (75–100%), the 500 nm also contributed to the emission. From the EL spectra, the emission came mainly from the polymer emitter even at a low PNP(1,4)-TF concentration in PVK (6 wt%). The J–V curves (left axis) of the OLED devices (Figure 5b) exhibited a shift to the lower turn-on voltage as the weight percentage of PNP(1,4)-TF in PVK increased from 0% (pure PVK) to 100% (pure PNP(1,4)-TF). Especially, in a high weight ratio (75–100%), the turn-on voltage had a dramatic decrement from ~8 V (0–45%) to about 4 V. The luminance is also plotted in Figure 5b (right axis), and the maximum value of the luminance for each concentration and its corresponding voltage are summarized in Table S4. As the concentration of PNP(1,4)-TF increased from 0 wt%, the maximum luminance with a peak value of 456 cd m^−2^ at 11.0 V was obtained for the blend with a 45% PNP(1,4)-TF in PVK. Increasing the polymer concentration was also found to lower the working voltage range and the voltage where the maximum luminance is observed. The luminance efficiency and the EQE of the same set of devices are calculated and plotted in Figure 5c,d, respectively. The data are tabulated in Table S4. Interestingly, the luminance efficiency (0.16 cd A^−1^) and the EQE (0.25%) were highest for the 6 wt% PNP(1,4)-TF:PVK OLEDs. Unlike the polymer PNP(1,4)-PT, the addition of PNP(1,4)-TF to PVK could simultaneously enhance the luminance efficiency and the EQE by 4–5 times. To see if the EQE could be higher, we measured the absolute photoluminescence quantum yield (PLQY) to estimate the theoretical maximum EQE values. The details can be found in Supplementary Materials. It indicates that the PLQY of PNP(1,4)-PT:PVK was around 2–7%, which indicates that theoretical maximum EQE is 0.1–0.35%. However, for PNP(1,4)-TF:PVK, the PLQY is highest (38%) at 6 wt% doping concentration, which demonstrates the theoretical maximum EQE of up to 1.9%. Therefore, we optimized the device structure by depositing TPBi as the electron transport layer (ETL) in between the emitting layer and the electrode. As shown in Figure 6, the highest EQE value was measured to be ~1% and a luminance efficiency of ~1.1 Cd A^−1^ could be achieved for this blue emitter in a device structure of ITO/PEDOT:PSS/PNP(1,4)-TF:PVK/TPBi/LiF/Al for 6 wt% doping concentration.

The AFM images of PNP(1,4)-TF:PVK films are shown in Figure S7. The film roughness is tabulated in Table S5 in Supplementary Materials. The pristine PNP(1,4)-TF and PVK films showed a roughness of ~0.8 nm. Other films, including the 6 wt% blend film, showed a higher roughness, i.e., the addition of PNP(1,4)-TF to PVK was also observed to enhance the film roughness, as observed for the polymer PNP(1,4)-PT. However, the roughness of PNP(1,4)-TF:PVK films was lower than that of PNP(1,4)-PT:PVK films, indicating better electrons and holes transport to the emitting layer.

#### 3.6.3. Discussion

In both the polymer blend systems, a higher efficiency was obtained for the blend where the percentage of emitting polymer was <10% in the blend. Assuming that the emitting polymer was uniformly dispersed in PVK, the higher efficiency for the given architecture can be attributed to two factors: the efficient radiative recombination and the favorable film morphology. The morphology, studied using AFM, indicates that the blend films were rougher than the pristine films. However, the devices fabricated with pristine films showed lower luminance and efficiency. This suggests that exciton–exciton quenching is one of the dominant loss mechanisms in PNP-based films. The monomolecular PL decay in both the materials (in diluted solution) was less than 1 ns (Figure S8) and the PL decay often showed even smaller decay time in the case of pristine films [38]. Nano-second transient photoluminescence analyses of blend polymer system have shown that the dispersion of emitting polymer in a larger band gap polymer can reduce the exciton–exciton quenching, resulting in an efficient charge recombination. Hence, results in the current study demonstrate that the dispersion of PNP(1,4)-PT and PNP(1,4)-TF in a larger band gap PVK resulted in lowering of exciton–exciton quenching and better charge recombination.

## 4. Conclusions

To summarize, three new copolymers based on 1,4-naphthalene (NP) were designed and synthesized by selecting phenothiazine (PT), triphenylamine substituted fluorene (TF), and anthanthrene (ANT), respectively, as the comonomers. From a molecular structure perspective, PNP(1,4)-TF, bearing perpendicular aryl side groups, exhibits a highly twisted structural geometry, which enables it not only a very good solubility but also a quite high thermal stability in comparison with other two polymers. In PVK-host-based OLED devices, the addition of 6 wt% PNP(1,4)-TF simultaneously improved the luminance efficiency and the EQE (up to 1% by inserting the TPBi ETL in the regular device structure) with a blue-color emitting. Overall, this structural geometry study of copolymers based on 1,4-naphthalene demonstrates that the PNP(1,4)-TF with twisted backbone and side-chain geometries emerges as a promising guest material for blue-color OLEDs.

## Figures and Tables

**Table 1:** Extracted data from the UV-Vis, PL spectra, and PESA curves (* S0→S1 transition).

**Polymer**	$\lambda_{max.}^{soln}$	$\lambda_{PL}^{soln}$	$\lambda_{max.}^{film}$	$\lambda_{onset}^{film}$	$E_{g}^{opt.}$	HOMO/LUMO
PNP(1,4)-PT	332 nm	496 nm	339 nm	440 nm	2.82 eV	−5.55/−2.73 eV
PNP(1,4)-TF	310 nm 363 * nm	426 nm	310 nm	413 nm	3.00 eV	−5.81/−2.81 eV
PNP(1,4)-ANT	327 nm 461 * nm	474 nm 502 * nm	327 nm	483 nm	2.57 eV	−5.53/−2.96 eV

## Data Availability

Data sharing not applicable.

## Supplementary Materials

The following are available online at https://www.mdpi.com/article/10.3390/polym13172914/s1. Scheme S1: The synthetic routes to the three polymers, PNP(1,4)-PT, PNP(1,4)-TF, and PNP(1,4)-ANT; Table S1: Extracted data from DFT computed absorption and emission spectra; Table S2: Summary of PNP(1,4)-PT:PVK devices; Table S3: Summary of roughness of PNP(1,4)-PT:PVK films; Table S4: Device characteristics of OLEDs based on PNP(1,4)-TF/PVK blends; Table S5: Summary of roughness of PNP(1,4)-TF:PVK films; Table S6: PLQY for the dispersed (6–75 wt% doping concentration) and pristine films. Figure S1: The (**a**–**c**) TGA and (**d**) DSC thermograms for the three polymers measured under nitrogen atmosphere. Heating rate: 10 °C min^−1^; Cooling rate: 5 °C min^−1^; Figure S2: The solution appearance of three polymers (~1.0 mg mL^−1^ in chloroform) under visible (left of each polymer) and UV (365 nm, right of each polymer) light; Figure S3: DFT computed (**a**) absorption and (**b**) emission spectra of polymer dimers with short side chains in replacement of the long chains; Figure S4: PNP(1,4)-PT pristine device at (**a**) a low voltage or (**b**) a high voltage, (**c**) A PNP(1,4)-PT:PVK (75 wt%) device showing a blue-green emission; Figure S5: The AFM images of ITO/PEDOT:PSS/PNP(1,4)-PT: PVK films. The weight percentages of PNP(1,4)-PT in PVK are (**a**) 100%, (**b**) 45%, (**c**) 18%, (**d**) 6%, and (**e**) 0%, respectively; Figure S6: (**a**) PNP(1,4)-TF pristine device operated at a low voltage, (**b**) a PNP(1,4)-TF:PVK (75 wt%) device operated at a high voltage, (**c**) a pristine PVK device operated at a high voltage; Figure S7: The AFM images of ITO/PEDOT:PSS/PNP(1,4)-TF: PVK films. The weight percentages of PNP(1,4)-TF in PVK are (**a**) 100%, (**b**) 75%, (**c**) 45%, (**d**) 18%, (**e**) 6%, and (**f**) 0%, respectively; Figure S8: The TCSPC spectra of dilute solution of (**a**) PNP(1,4)-PT and (**b**) PNP(1,4)-TF in *p*-xylene; Figure S9: ^1^H NMR spectrum of BE-NP(1,4); Figure S10: ^13^C NMR spectrum of BE-NP(1,4); Figure S11: ^1^H NMR spectrum of BE-PT; Figure S12: ^1^H NMR spectrum of DB-TF; Figure S13: ^13^C NMR spectrum of DB-TF; Figure S14: ^1^H NMR spectrum of DB-ANT.
