# Supplementary material for: Structural Geometry Variation of 1,4-Naphthalene-Based Co-Polymers to Tune the Device Performance of PVK-Host-Based OLEDs
Source: Polymers (Basel). 2021 Aug 30;13(17):2914. doi: 10.3390/polym13172914 (PMC8434216; doi:10.3390/polym13172914)
Supplement: Supplementary file 1 [file polymers-13-02914-s001.zip › polymers-1286857-supplementary.pdf]

Supplementary Materials

**Experimental Section**	**Page S-3**
**Scheme S1.** The synthetic routes to the three polymers, PNP(1,4)-PT, PNP(1,4)-TF, and PNP(1,4)-ANT.	**Page S-3**
**Figure S1.** The (a-c) TGA and (d) DSC thermograms for the three polymers measured under nitrogen atmosphere. Heating rate: 10 ^oC min^{-1}; Cooling rate: 5 ^oC min^{-1}.	**Page S-7**
**Figure S2.** The solution appearance of three polymers (~1.0 mg mL^{-1} in chloroform) under visible (left of each polymer) and UV (365 nm, right of each polymer) light.	**Page S-8**
**Figure S3.** DFT computed (a) absorption and (b) emission spectra of polymer dimers with short side chains in replacement of the long chains.	**Page S-9**
**Table S1.** Extracted data from DFT computed absorption and emission spectra.	**Page S-10**
**Figure S4.** PNP(1,4)-PT pristine device at (a) a low voltage or (b) a high voltage; (c) A PNP(1,4)-PT:PVK (75 wt%) device showing a blue-green emission.	**Page S-11**
**Table S2.** Summary of PNP(1,4)-PT:PVK devices.	**Page S-12**
**Figure S5.** The AFM images of ITO/PEDOT:PSS/PNP(1,4)-PT: PVK films. The weight percentages of PNP(1,4)-PT in PVK are (a) 100%, (b) 74%, (c) 45%, (d) 18%, (e) 6%, and (f) 0%, respectively.	**Page S-13**
**Table S3.** Summary of roughness of PNP(1,4)-PT:PVK films.	**Page S-14**
**Figure S6.** (a) PNP(1,4)-TF pristine device operated at a low voltage; (b) A PNP(1,4)-TF:PVK (75 wt%) device operated at a high voltage; (c) A pristine PVK device operated at a high voltage.	**Page S-15**
**Table S4.** Device characteristics of OLEDs based on PNP(1,4)-TF/PVK blends.	**Page S-16**

**Figure S7.** The AFM images of ITO/PEDOT:PSS/PNP(1,4)-TF: PVK films. The weight percentages of PNP(1,4)-TF in PVK are (a) 100%, (b) 75%, (c) 45%, (d) 18%, (e) 6%, and (f) 0%, respectively.	**Page S-17**
**Table S5.** Summary of roughness of PNP(1,4)-TF:PVK films.	**Page S-18**
**Table S6.** PLQY for the dispersed (6-75 wt% doping concentration) and pristine films.	**Page S-19**
**Figure S8.** The TCSPC spectra of dilute solution of (a) PNP(1,4)-PT and (b) PNP(1,4)-TF in p-xylene.	**Page S-20**
**Figure S9.** ^{1}H NMR spectrum of BE-NP(1,4).	**Page S-21**
**Figure S10.** ^{13}C NMR spectrum of BE-NP(1,4).	**Page S-22**
**Figure S11.** ^{1}H NMR spectrum of BE-PT.	**Page S-23**
**Figure S12.** ^{1}H NMR spectrum of DB-TF.	**Page S-24**
**Figure S13.** ^{13}C NMR spectrum of DB-TF.	**Page S-25**
**Figure S14.** ^{1}H NMR spectrum of DB-ANT.	**Page S-26**
**References**	**Page S-27**

Experimental Section

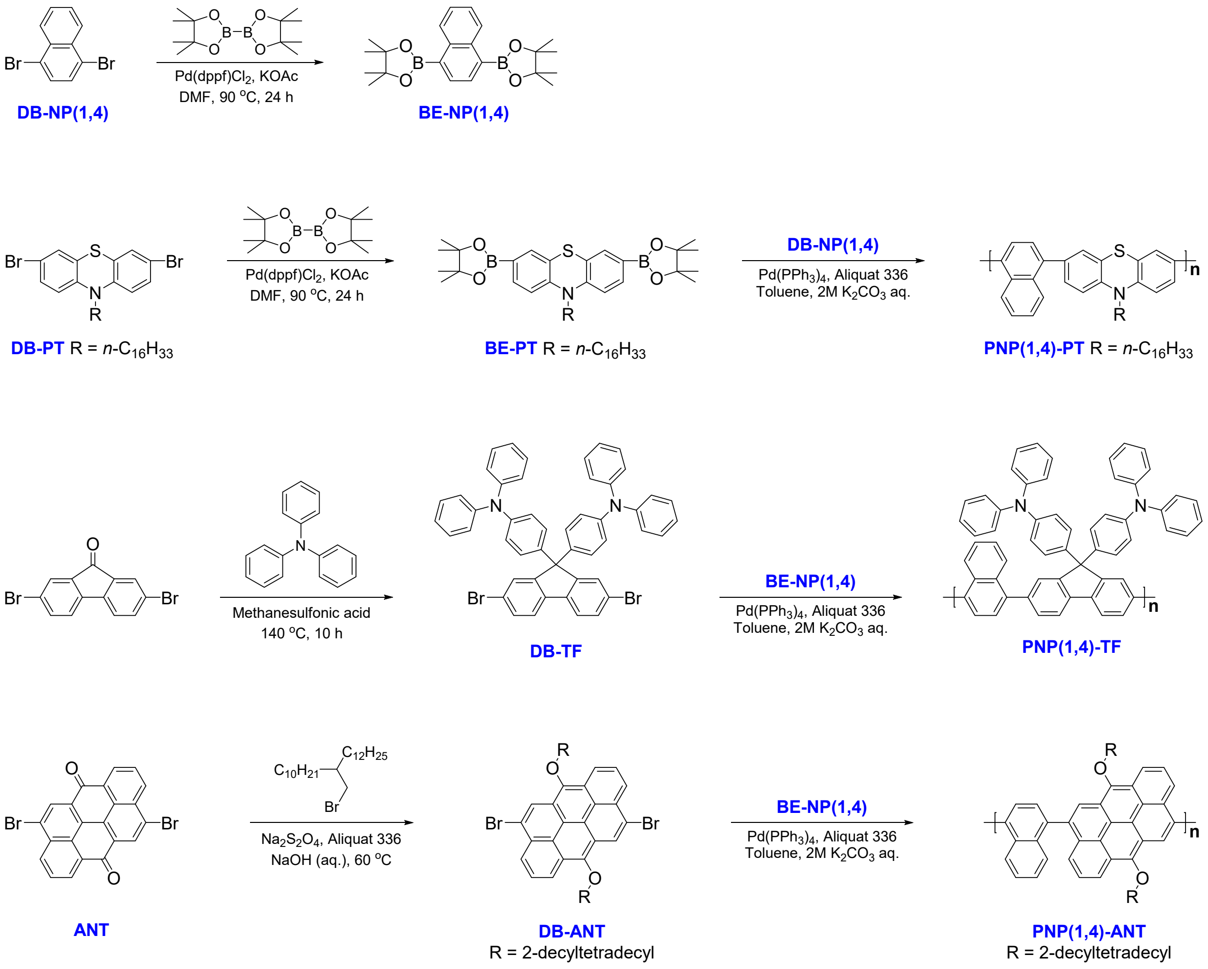

Scheme S1. The synthetic routes to the three polymers, PNP(1,4)-PT, PNP(1,4)-TF, and PNP(1,4)-ANT.

Unless otherwise stated, the starting compounds and solvents are commercially available (Sigma-Aldrich, Bide Pharmatech, Chem Supply, Alfa Aesar, Thermo Fisher Scientific, *et al.*) and used directly without further purification. The synthesized materials are characterized by ^1H and ^{13}C NMR (nuclear magnetic resonance) spectra which are recorded on Bruker 400 or 600 MHz NMR spectrometer with chloroform-d as the solvent and tetramethyl silane (TMS) as the internal standard. The molecular weights of polymers are measured by gel permeation chromatography (GPC) with a JASCO GULLIVER 1500 equipped with a pump (PU-2080 Plus), an absorbance

detector (RI-2031 Plus), and two Shodex GPC KF-803 columns (8.0 mm I.D. × 300 mm L). Tetrahydrofuran (THF) was used as the carrier solvent at the flow rate of 1.0 mL min^{-1}. The molecular weights were calculated based on a conventional calibration curve using polystyrene standards.

Synthesis of BE-NP(1,4)[1]: Under argon protection, DB-NP(1,4) (1.0 g, 3.5 mmol), bis(pinacolato)diboron (2.13 g, 8.4 mmol), Pd(dppf)Cl_2 (256 mg, 0.35 mmol), anhydrous KOAc (2.06 g, 21 mmol) and dry DMF (40 mL) are put together in a 100 mL round-bottom flask and heated up to 90 °C for 24 hours with vigorous stirring. After being cooled down to room temperature, the mixture is diluted with ethyl acetate and poured into water. The water layer is extracted with ethyl acetate three times and the combined organic phase is dried with Na_2SO_4. After removing the solvent, the residue is purified by running column with hexane: ethyl acetate = 10:1 as the eluent, yielding 1.0 g (~75%) of BE-NP(1,4) as a white power. ^{1}H NMR (400 MHz, $CDCl_3$) δ (ppm) 8.81 (m, 2H), 8.08 (s, 2H), 7.57 (m, 2H), 1.46 (s, 24H). ^{13}C NMR (101 MHz, $CDCl_3$) δ (ppm) 136.57, 134.36, 128.66, 125.90, 83.85, 24.99.

Synthesis of BE-PT: DB-PT was synthesized by following reported procedures.[2] Under argon protection, DB-PT (1.16 g, 2.0 mmol), bis(pinacolato)diboron (1.22 g, 4.8 mmol), Pd(dppf)Cl_2 (146 mg, 0.2 mmol), anhydrous KOAc (1.18 g, 12.0 mmol) and dry DMF (60 mL) are put together in a 100 mL round-bottom flask and heated up to 90 °C for 24 hours with vigorous stirring. After being cooled down to room temperature, the mixture is diluted with ethyl acetate and poured into water. The water layer is extracted with ethyl acetate three times and the combined organic phase is dried with Na_2SO_4. After removing the solvent, the residue is purified by running column with hexane:

dichloromethane = 1:1 as the eluent, yielding 1.0 g (~74%) of BE-PT as a pale yellow solid. ^{1}H NMR (600 MHz, $CDCl_3$) δ (ppm) 7.47 (dd, J = 8.1, 1.2 Hz, 2H), 7.44 (d, J = 1.4 Hz, 2H), 6.72 (d, J = 8.2 Hz, 2H), 3.76 (t, J = 6.8 Hz, 2H), 1.69 (m, 2H), 1.32 (m, 2H), 1.24 (s, 24H), 1.21-1.13 (m, 24H), 0.80 (m, 3H).

Synthesis of DB-TF:[3] Under argon protection, 2,7-dibromo-fluoren-9-one (1.01 g, 3 mmol), triphenylamine (12.25 g, 50 mmol) and methanesulfonic acid (1 mL) are mixed in a round-bottom flask. The mixture is heated up to 140 ^oC and stirred for 10 hours. After the reaction is cooled down to room temperature, the resulting mixture is dissolved and extracted with dichloromethane. The extracted solution is then washed twice with water and dried with Na_2SO_4. Evaporation of the solution gives a blue solid that is purified by running column with hexane: dichloromethane = 5:1 as the eluent, and then recrystallized to obtain the pure product DB-TF as a white solid (1.55 g, 64%). ^{1}H NMR (600 MHz, $CDCl_3$) δ (ppm) 7.48 (d, J = 7.8 Hz, 2H), 7.44 (d, J = 1.8 Hz, 2H), 7.39 (dd, J = 1.8, 7.8 Hz, 2H), 7.15 (t, J = 7.8 Hz, 8H), 7.00 (d, J = 7.8 Hz, 8H), 6.91 (m, 8H), 6.84 (m, 4H). ^{13}C NMR (151 MHz, $CDCl_3$) δ (ppm) 153.49, 147.54, 146.76, 138.00, 137.68, 130.84, 129.40, 129.29, 128.71, 124.70, 123.08, 122.79, 121.79, 121.57, 64.67.

Synthesis of DB-ANT:[4] Under argon protection, ANT (1 g, 2.15 mmol), $Na_2S_2O_4$ (940 mg, 5.4 mmol), Aliquat 336 (1.15 g, 2.58 mmol), aqueous NaOH (0.1 M, 100 mL, 10 mmol) and 2-decyltetradecyl bromide (7.18 g, 17.2 mmol) are added in a 250 mL round flask. The mixture is heated up to 60 ^oC and kept stirring overnight. When the temperature is cooled down to room temperature, the water is decanted, and methanol is added. The crude product is filtered and washed with methanol. The pure product

DB-ANT (1.5 g, 1.32 mmol, yield = 61%) as an orange solid can be afforded by running column with hexane as the eluent. ^{1}H NMR (600 MHz, $CDCl_3$) δ (ppm) 8.58 (m, 6H), 8.12 (d, J = 7.8 Hz, 2H), 4.13 (m, 4H), 2.11 (m, 2H), 1.86 (m, 4H), 1.70 (m, 4H), 1.59 - 1.32 (m, 72H), 0.92 (m, 12H).

Thermal Properties: Thermal stability was measured using a Pegasus Q500 TGA thermogravimetric analyser under nitrogen atmosphere at a heating rate of 10 ^{o}C min^{-1} from 20 ^{o}C to 800 ^{o}C. Differential scanning calorimetry (DSC) measurements were conducted under nitrogen atmosphere using a Chimaera instrument Q100 DSC at a heating rate of 10 ^{o}C min^{-1} and cooling rate of 5 ^{o}C min^{-1} between 20 and 250 ^{o}C.

Optical Properties: UV-Vis absorption and fluorescence emission were recorded using Varian Cary 60 and Eclipse spectrophotometers, respectively. Photoelectron spectroscopy in air (PESA) measurements were conducted using on an AC-2 photoelectron spectrometer (Riken-Keiki Co.).

DFT Calculations: Density functional theory (DFT)[5] calculations were performed in Gaussian 16[6] using the B3LYP[7] functional and the 6-31+g(d,p) basis set. PCM (polarizable continuum) model[8] of the chloroform solvent was used. Optical properties were computed with Time-Dependent DFT[9] using 10 lowest singlet excited states.

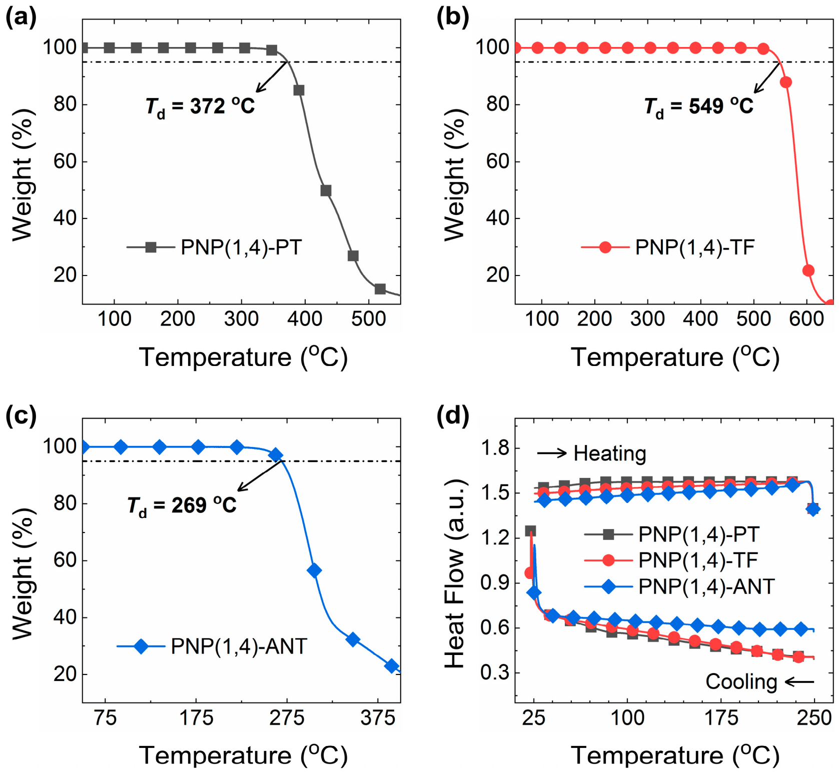

Figure S1. The (a-c) TGA and (d) DSC thermograms for the three polymers measured under nitrogen atmosphere. Heating rate: 10 °C min^{-1}; Cooling rate: 5 °C min^{-1}.

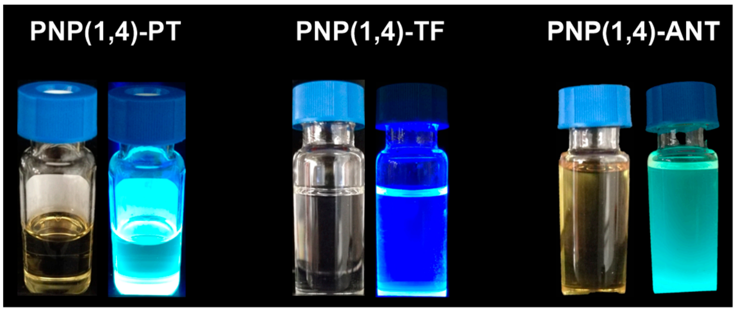

Figure S2. The solution appearance of three polymers (~1.0 mg mL^{-1} in chloroform) under visible (left of each polymer) and UV (365 nm, right of each polymer) light.

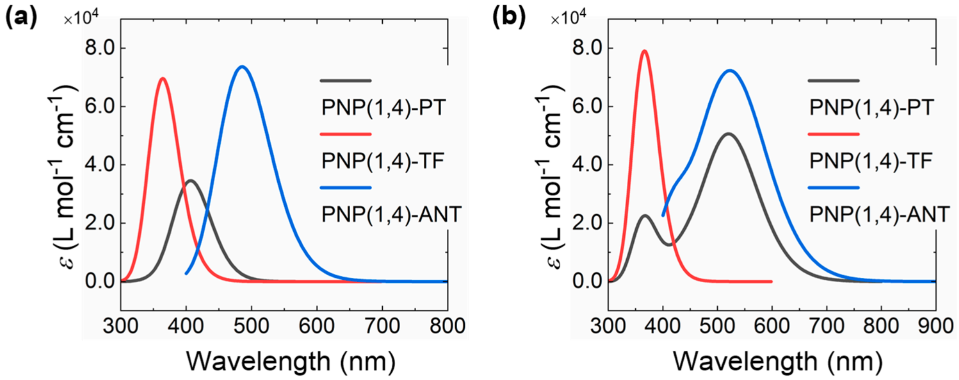

Figure S3. DFT computed (a) absorption and (b) emission spectra of polymer dimers with short side chains in replacement of the long chains.

Table S1. Computed absorption and emission spectra and comparison with experimental data.

Polymer	$\lambda_{max.}^{abs.}$	$\lambda_{max.}^{PL}$	Stokes Shift	
PNP(1,4)-PT	404 nm	518 nm	114 nm [a)]	(496-332) 164 nm [b)]
PNP(1,4)-TF	364 nm	365 nm	1 nm [a)]	(426-363) 63 nm [b)]
PNP(1,4)-ANT	485 nm	522 nm	37 nm [a)]	(502-461) 41 nm [b)]

[a)] DFT results; [b)] Experimental results.

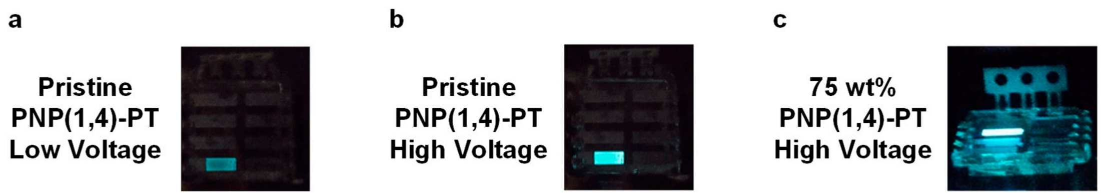

Figure S4. PNP(1,4)-PT pristine device at (a) a low voltage or (b) a high voltage; (c) A PNP(1,4)-PT:PVK (75 wt%) device showing a blue-green emission.

Table S2. Summary of PNP(1,4)-PT:PVK devices.

PNP(1,4)-PT wt% in PVK	EL Max (nm)	L_{max} (cd m^{-2}, @V)		L_{effi} (cd A^{-1}, @V)		EQE Max (%, @V)	
100	500	46	(8.4V)	0.004	(8.4V)	<0.001	(8 V)
45	495	46	(9 V)	0.02	(9V)	0.007	(9 V)
18	495	44	(10 V)	0.04	(10 V)	0.014	(10 V)
**6**	**492**	**583**	**(11 V)**	**0.2**	**(10 V)**	**0.06**	**(10 V)**
0 (PVK)	392	71	(10.5 V)	0.03	(10 V)	0.07	(10 V)

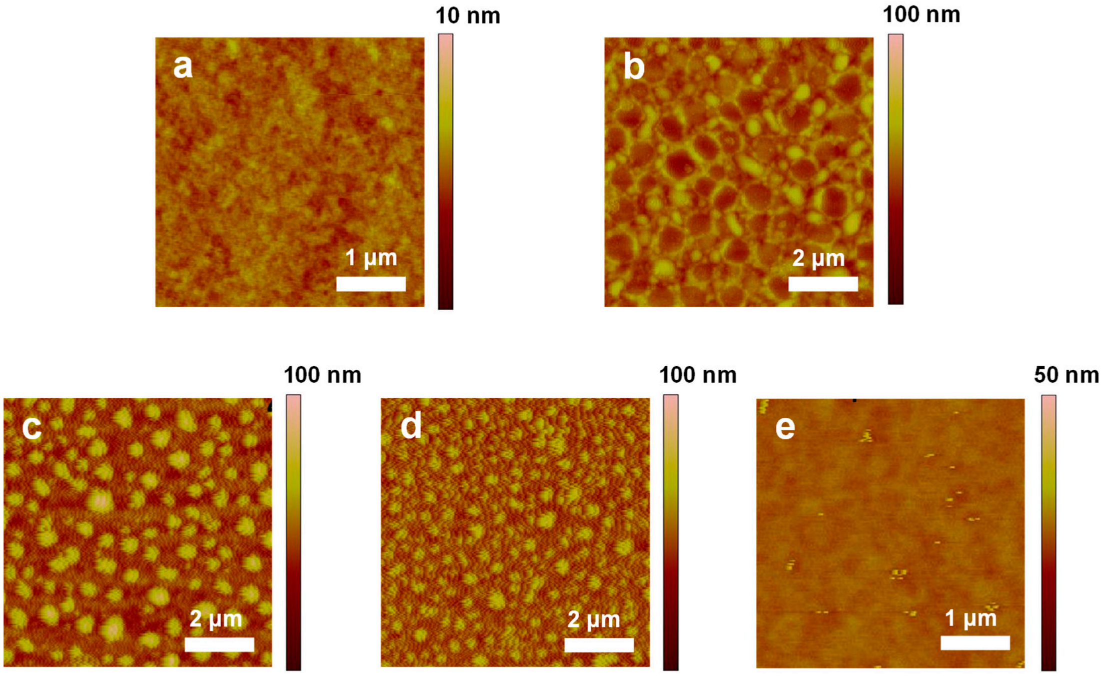

Figure S5. The AFM images of ITO/PEDOT:PSS/PNP(1,4)-PT: PVK films. The weight percentages of PNP(1,4)-PT in PVK are (a) 100%, (b) 45%, (c) 18%, (d) 6%, and (e) 0%, respectively.

Table S3. Summary of roughness of PNP(1,4)-PT:PVK films.

Entry	wt% of PNP(1,4)-PT in PVK	Roughness
a	100% [PNP(1,4)-PT]	0.552 nm
c	45%	6.98 nm
d	18%	10.4 nm
e	6%	7.12 nm
f	0% [PVK]	1.28 nm

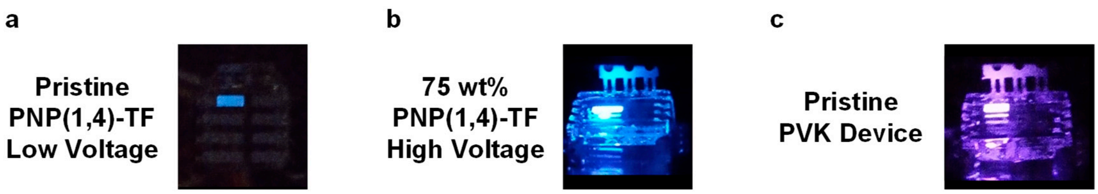

Figure S6. (a) PNP(1,4)-TF pristine device operated at a low voltage; (b) A PNP(1,4)-TF:PVK (75 wt%) device operated at a high voltage; (c) A pristine PVK device operated at a high voltage.

Table S4. Device characteristics of OLEDs based on PNP(1,4)-TF/PVK blends.

PNP(1,4)-TF wt% in PVK	EL Max (nm)	L_{max} (cd m^{-2}, @V)		L_{effi} (cd A^{-1}, @V)		EQE Max (%, @V)	
100%	430, 486	85	(6.8 V)	0.008	(6.8 V)	0.004	(6.8 V)
75%	430, 483	144	(7.0 V)	0.015	(5.8 V)	0.011	(5.8 V)
45%	420, 482	456	(11.0 V)	0.09	(8.6 V)	0.1	(8.6 V)
18%	425, 482	234	(11.0 V)	0.09	(9.0 V)	0.12	(9.0 V)
**6%**	**420, 482**	**289**	**(11.4 V)**	**0.16**	**(7.0 V)**	**0.25**	**(7.0 V)**
PVK	392	71	(10.5 V)	0.03	(10.0 V)	0.07	(10.0 V)

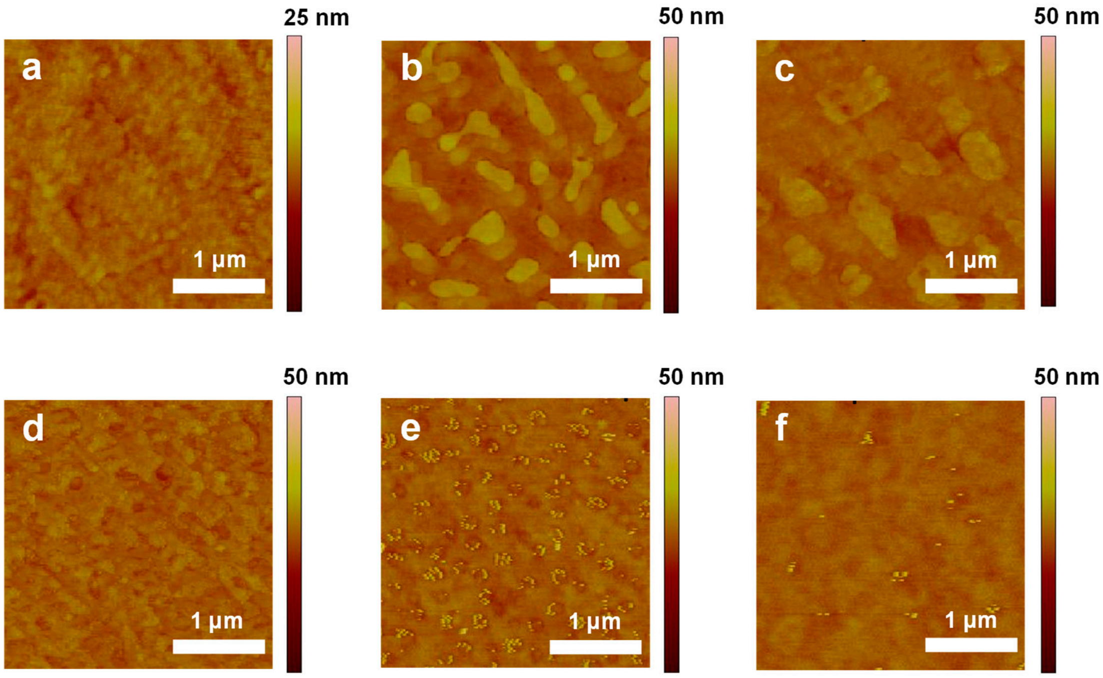

Figure S7. The AFM images of ITO/PEDOT:PSS/PNP(1,4)-TF: PVK films. The weight percentages of PNP(1,4)-TF in PVK are (a) 100%, (b) 75%, (c) 45%, (d) 18%, (e) 6%, and (f) 0%, respectively.

Table S5. Summary of roughness of PNP(1,4)-TF:PVK films.

Entry	wt% of PNP(1,4)-TF in PVK	Roughness
a	100% [PNP(1,4)-TF]	0.808 nm
b	75%	2.38 nm
c	45%	1.44 nm
d	18%	1.44 nm
e	6%	2.14 nm
f	0% [PVK]	1.28 nm

We measured the absolute photoluminescence quantum yield (PLQY, γ_{PLQY}) of the doped and pristine films since it is related to the OLED EQE as:

$$EQE = \gamma_{PLQY} \times \eta_{S/T} \times \eta_{CC} \times \eta_{OC}$$

where, the maximum theoretical values of singlet generation yield, $\eta_{S/T}$, is 25%; charge carrier balance, η_{CC}, is 100% and outcoupling efficiency, η_{OC} is 20%. For PNP(1,4)-PT, the PLQY value (2 - 7%) limits the theoretical max. EQE value to 0.1 - 0.35%. For PNP(1,4)-TF, the PLQY is the highest (38%) for 6 wt% doping concentration and thus the theoretical max. EQE is 1.9%. The PLQY is almost the same (23%) as that of the pristine film for higher doping concentrations. Therefore, we fabricated OLED with an ETL (TPBi) in between the emitter layer and the cathode for PNP(1,4)-TF with 6 wt% doping in PVK and could achieve EQE of ~1% and luminance efficiency of ~1.1 Cd A^{-1} in a device configuration of ITO/PEDOT:PSS/PNP(1,4)-TF:PVK/TPBi/LiF/Al. The result of the same is included as Figure 6 in the main text.

Table S6: PLQY for the dispersed (6-75 wt% doping concentration) and pristine films.

	**6%**	**18%**	**45%**	**75%**	**pristine**
PNP(1,4)-TF	38%	26%	25%	24%	23%
PNP(1,4)-PT	2%	3%	7%	6%	4%

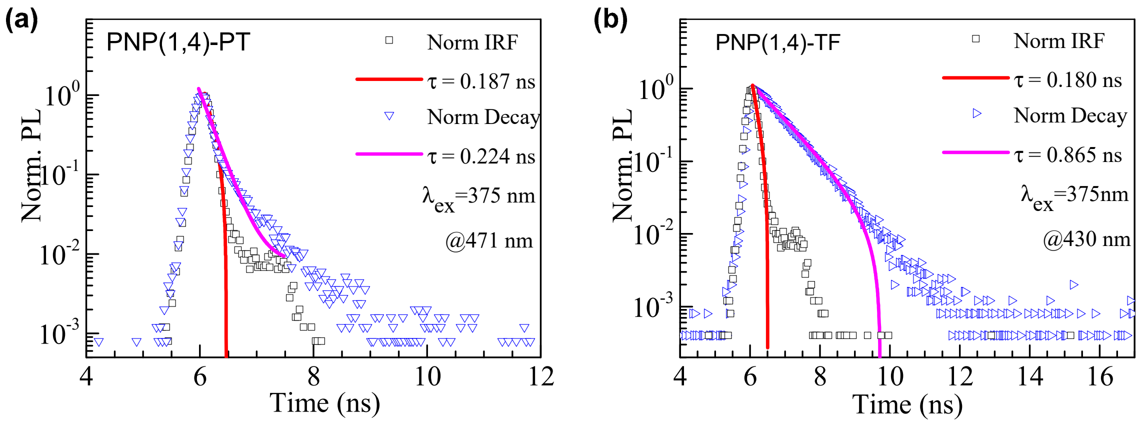

Figure S8. The TCSPC spectra of dilute solution of (a) PNP(1,4)-PT and (b) PNP(1,4)-TF in p-xylene.

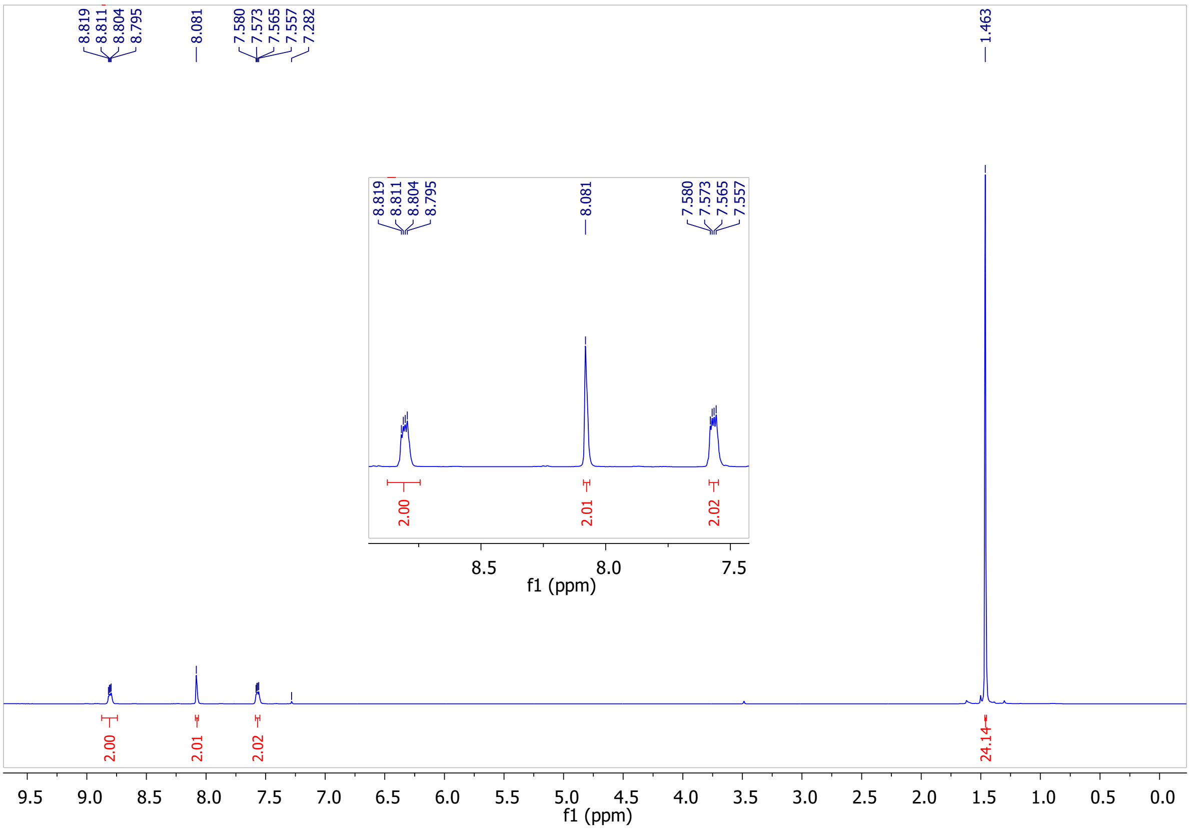

Figure S9. ^{1}H NMR spectrum of BE-NP(1,4).

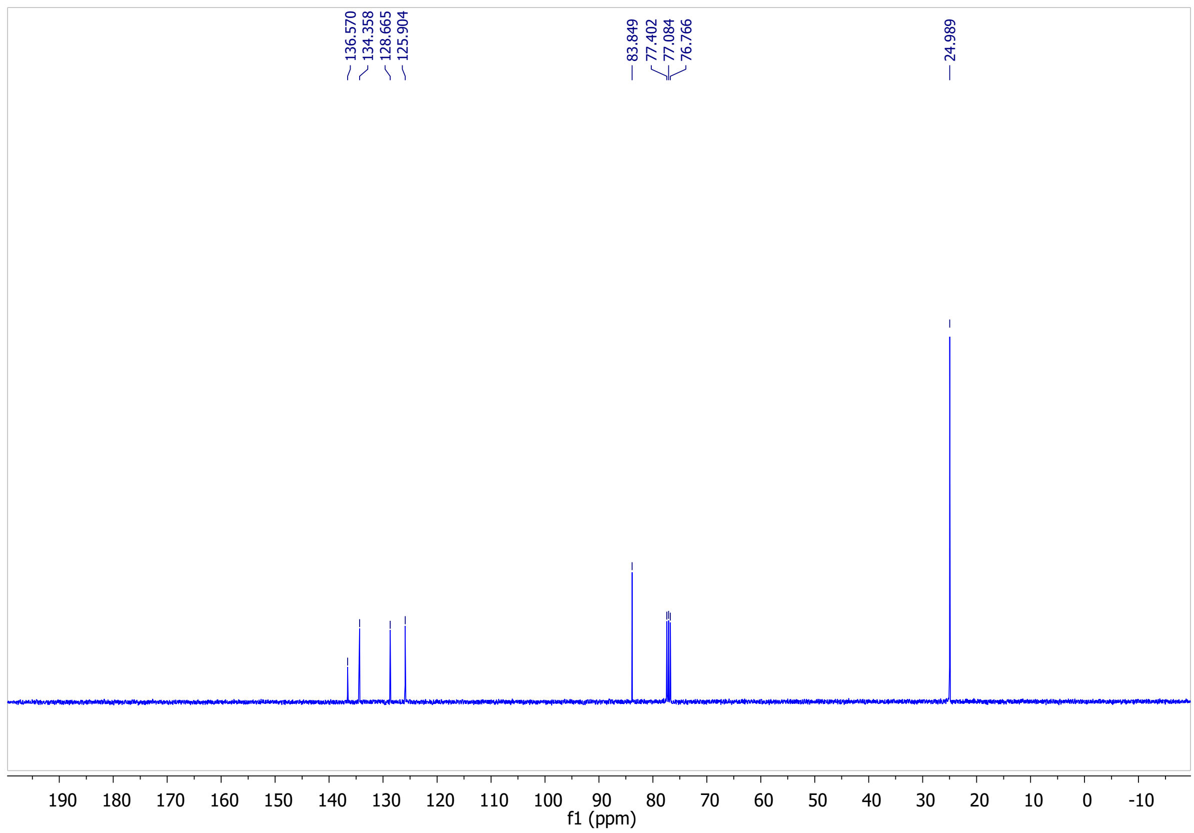

Figure S10. ^{13}C NMR spectrum of BE-NP(1,4).

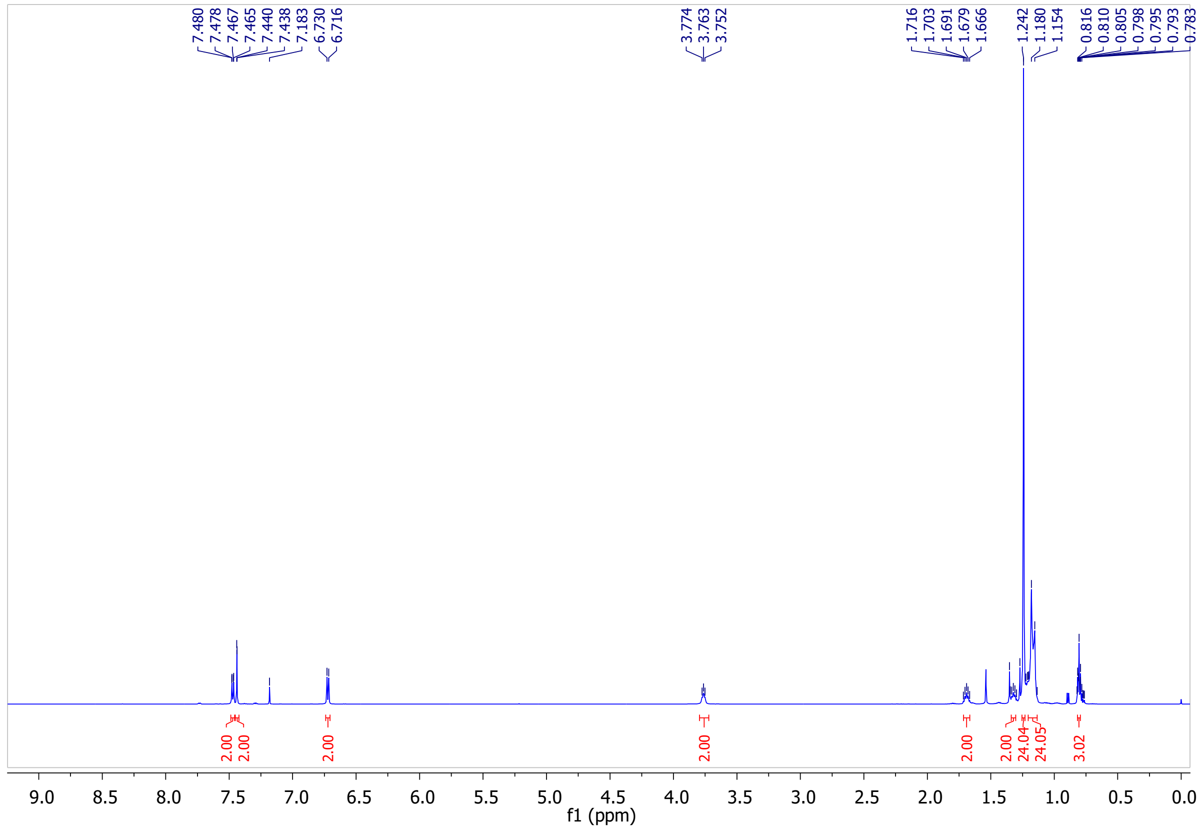

Figure S11. ^{1}H NMR spectrum of BE-PT.

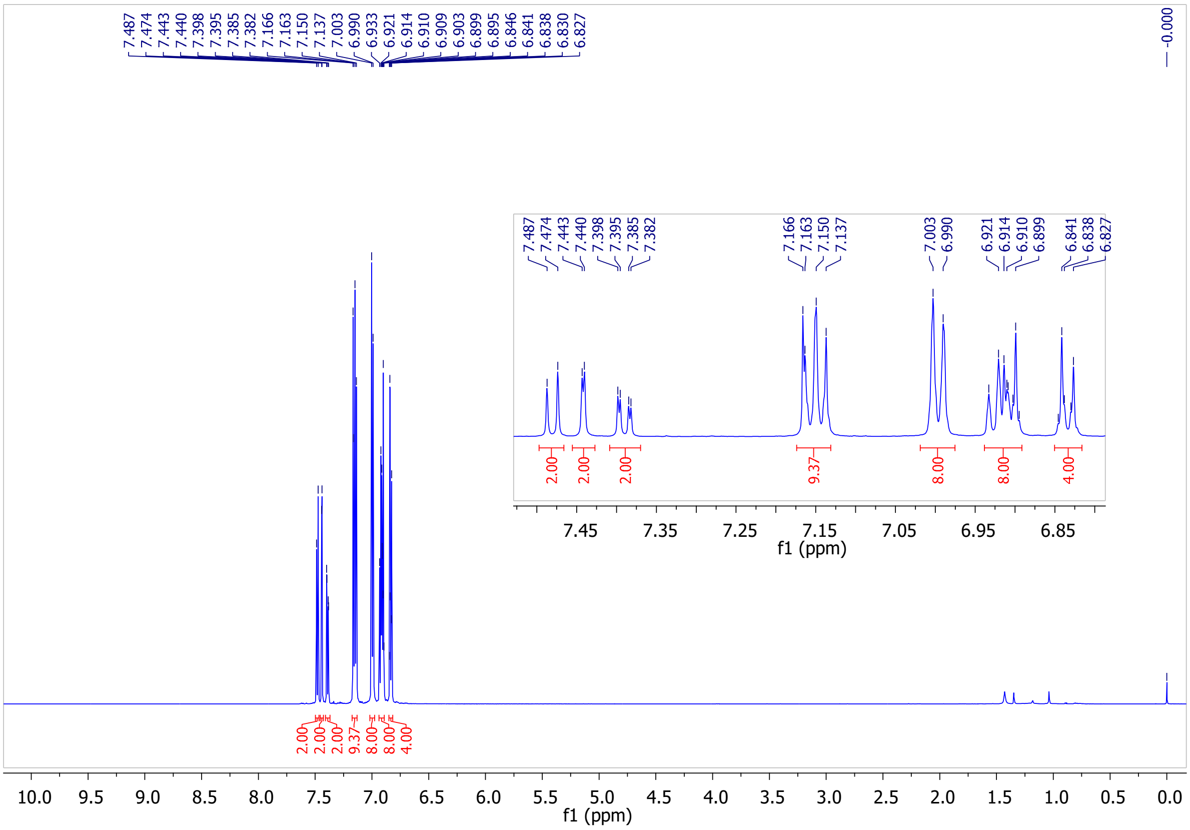

Figure S12. ^{1}H NMR spectrum of DB-TF.

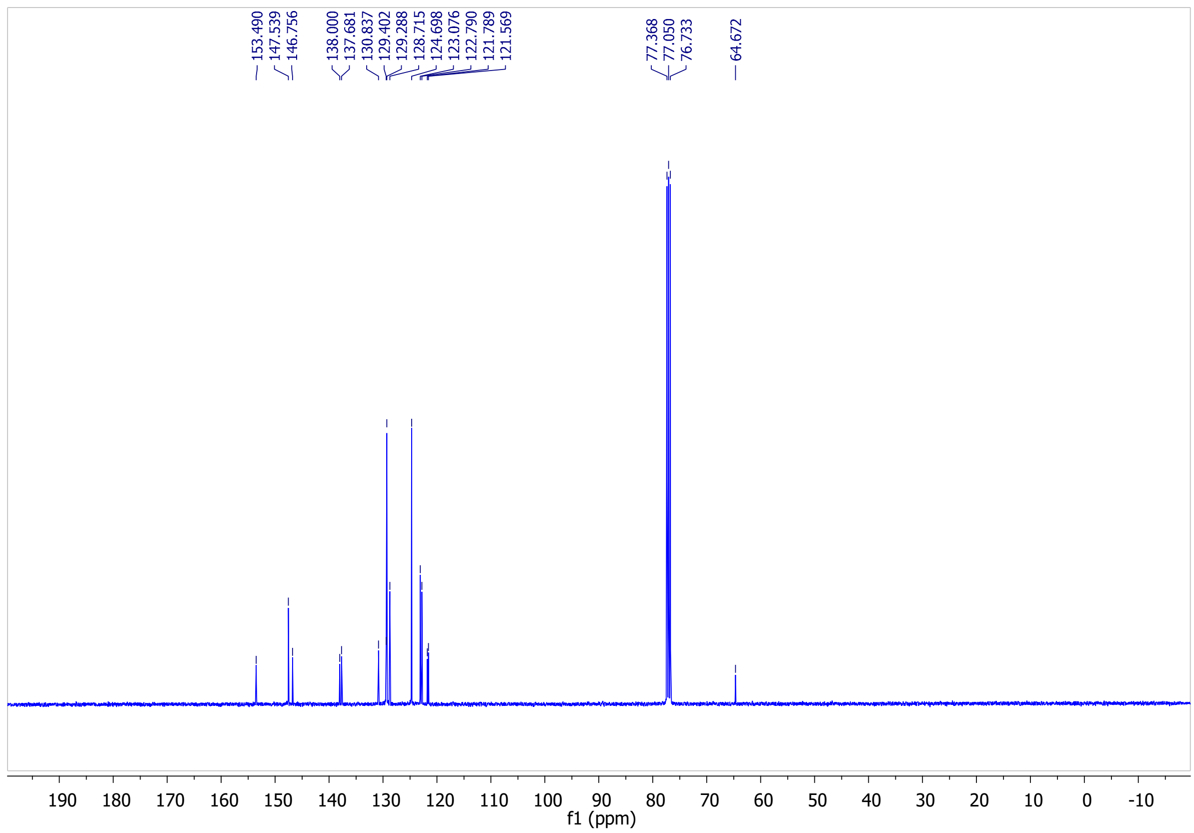

Figure S13. ^{13}C NMR spectrum of DB-TF.

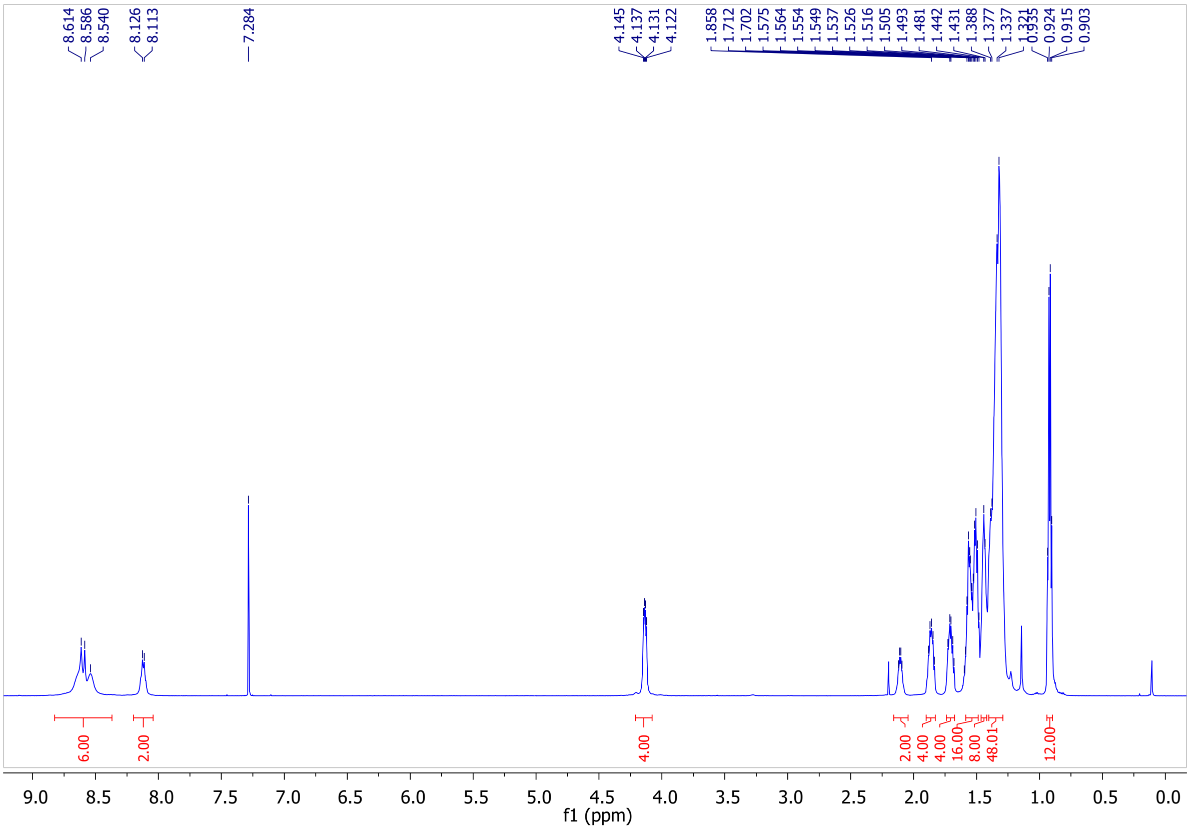

Figure S14. ^{1}H NMR spectrum of DB-ANT.

References

1. D. Alezi, Y. Belmabkhout, M. Suyetin, P. M. Bhatt, Ł. J. Weseliński, V. Solovyeva, K. Adil, I. Spanopoulos, P. N. Trikalitis, A.-H. Emwas and M. Eddaoudi, *J. Am. Chem. Soc.*, 2015, **137**, 13308-13318.
2. W. Tang, T. Kietzke, P. Vemulamada and Z.-K. Chen, *J. Polym. Sci., Part A: Polym. Chem.*, 2007, **45**, 5266-5276.
3. E. Christophe, G. A. C., U. Frank, Y. Gang, S. Gordana and M. Klaus, *Adv. Mater.*, 2002, **14**, 809-811.
4. J.-B. Giguère, Q. Verolet and J.-F. Morin, *Chem. - Eur. J.*, 2013, **19**, 372-381.
5. W. Kohn and L. J. Sham, *Phys. Rev.*, 1965, **140**, A1133.
6. M. Frisch, G. Trucks, H. Schlegel, G. Scuseria, M. Robb, J. Cheeseman, G. Scalmani, V. Barone, B. Mennucci and G. Petersson, 2005.
7. A. D. Becke, *J. Chem. Phys.*, 1993, **98**, 5648-5652.
8. G. Scalmani and M. J. Frisch, *J. Chem. Phys.*, 2010, **132**, 114110.
9. C. Adamo and D. Jacquemin, *Chem. Soc. Rev.*, 2013, **42**, 845-856.
